# Reply to Detection of *Leptospira interrogans* DNA in Urine of a Captive Ocelot (*Leopardus pardalis*)

**DOI:** 10.3390/ijerph18020796

**Published:** 2021-01-19

**Authors:** Andrea Murillo, Rafaela Cuenca, Emmanuel Serrano, Goris Marga, Ahmed Ahmed, Salvador Cervantes, Cristina Caparrós, Verónica Vieitez, Andrea Ladina, Josep Pastor

**Affiliations:** 1 Department de Medicina i Cirurgia Animals, Facultat de Veterinària, Universitat Autònoma de Barcelona, Barcelona (UAB), CP 08193 Bellaterra, Spain; rafaela.cuenca@uab.es (R.C.); Josep.Pastor@uab.cat (J.P.); 2 Wildlife Ecology & Health Group (WE&H), Servei d’Ecopatologia de Fauna Salvatge (SEFaS), Universitat Autònoma de Barcelona (UAB), CP 08193 Bellaterra, Spain; emmanuel.serrano@uab.cat; 3 OIE and National Collaborating Centre for Reference and Research on Leptospirosis (NRL), Amsterdam UMC, University of Amsterdam, Medical Microbiology, Meibergdreef 39, 1105 AZ Amsterdam, The Netherlands; m.goris@amsterdamumc.nl (G.M.); a.ahmed@amsterdamumc.nl (A.A.); 4 Clínica Felina Barcelona, CP 08015 Barcelona, Spain; clinicafelinabarcelona@gmail.com (S.C.); cristinacapa94@gmail.com (C.C.); 5 Facultad de Veterinaria, Universidad de Extremadura, CP 10003 Cáceres, Spain; vvieitez@gmail.com (V.V.); rabacha@unex.es (A.L.)

In reply to a comment, we want to mention that we consider this report significant in animal leptospirosis. The presence of leptospiral DNA in the urine of a captive wild cat supports findings described in previous reports [1,2], as in our study, where leptospiral DNA is detected in urine without the presence of anti-leptospiral antibodies.

This finding further increases the likelihood that domestic and wild cats play a key role in the maintenance and transmission of *Leptospira* spp. The methodology used in this work was similar to ours. Concerning the MAT technique, we would like to mention that the panel of serovars used was wide and followed the suggestions of the World Organization for Animal Health. There are differences in some of the serovars used between studies, but this is normal. For the accurate diagnostic and epidemiological surveillance of leptospirosis, it is imperative to use endemic serovars in the geographic region.

Concerning the PCR methodology, it was not only used for the detection of the lipL32 gene (present only in pathogenic *Leptospira* spp.), one step further was taken, and a sequence that allowed the identification of the pathogenic *Leptospira* spp. as *Leptospira interrogans* was made.

In conclusion, we are aware that this type of research helps us to improve our knowledge about leptospirosis in terms of its epidemiology and reservoir animals.
